# Commentary on: Frequently asked questions on erectile dysfunction: evaluating artificial intelligence answers with expert mentorship

**DOI:** 10.1038/s41443-024-00901-x

**Published:** 2024-05-24

**Authors:** Nikit Venishetty, Omer A. Raheem

**Affiliations:** 1 Paul L. Foster School of Medicine, Texas Tech Health Sciences Center, El Paso, TX USA; 2 Department of Surgery, Section of Urology, The University of Chicago, Chicago, IL USA

**Keywords:** Biotechnology, Health care

In an article recently accepted for publication in the journal of International Journal of Impotence Research, Baturu et al. evaluated the accuracy of artificial intelligence (AI)-generated responses to frequently asked questions on erectile dysfunction (ED) [1]. By using two expert urologists who evaluated questions through the Global Quality Score (GQS), there were significant agreement measurements across AI platforms including BARD, ChatGPT 3.5, and ChatGPT 4 [1]. Specifically Baturu’s study found that ChatGPT 3.5 and ChatGPT 4 achieved a higher GQS compared to BARD in categories including causes (*p* < 0.001), treatment options (*p* < 0.001), protective measures (*p* < 0.013), relationships with other illnesses (*p* = 0.006) and treatment with herbal agents (*p* = 0.043). Moreover, the authors used the F1 metric to evaluate the models’s accuracy in machine learning (ML). With a higher score (1) indicating a better model performance, the authors found an overall F1 score of 0.58. Specific categories like causes, diagnosis, treatment options, and protective measures showed excellent results, while others lacked reliability due to the absence of information, warranting improvement in generated answers for those categories. The authors concluded that there was no significant difference between ChatGPT 3.5, ChatGPT 4, and BARD in terms of the quality of answers but had a better GQS.

It is apparent that over the past ten years, the use of AI in medicine has been evolving rapidly including machine learning (ML), artificial neural networks (ANNs), deep learning (DL), robots, and natural language processing (NLP) for massive data analysis. Specifically, AI chatbots have been increasingly used in various healthcare domains such as symptom detection to assist patients manage their conditions appropriately, with or without a physician [2, 3]. With more than 2.9 million outpatient visits made for ED, counsel, manage and treat their symptoms without invasive methodologies or delays in treatment [4]. Moreover, many men are embarrassed by their ED symptoms and experience concomitant (ED) in the United States alone, it becomes crucial to provide patients with an impactful way to shame, preventing them from reaching medical providers for assistance [5]. According to previous studies, only 32.4% of men feel comfortable in starting a conversation regarding ED with their providers [6]. Taken together, an impartial and unbiased entity such as ChatGPT may allow more men to seek help and care for their underlying ED and ultimately reducing significant anxiety associated with their medical issues. However, the use of AI language models has been understudied [7].

Current evidence has shown that AI responses can be used to provide valuable information regarding ED. Studies have found that AI open-source language models such as Google BARD and ChatGPT had significantly more accuracy, robustness, and unbiased responses compared to expert urologists [8]. Other studies have assessed the accuracy, readability, and reproducibility of ChatGPT’s answers to commonly requested questions about ED. The results demonstrated a fair degree of repeatability and high accuracy (Cohen’s kappa coefficient = 0.61) in providing thorough or accurate but insufficient answers about the epidemiology and dangers of ED [5]. On the other hand, comments about treatment and prevention were often too sophisticated for the average patient to read, and they were also less accurate with poor reliability [5].

It is important to note that GPT models are not trained in medical knowledge, while specialized systems such as Medpalm 2 can be used for medical purposes [9]. Another notable criticism of ChatGPT’s ability to assess medical inquiries is because of its human-like delivery style and propensity for patients to become unduly dependent on its responses without question, there is a significant risk of mistakes. Physicians must take an active role in the development and evaluation of AI-powered chatbots, rather than merely accepting them or interacting with them at a later stage, because of the unique character of these outputs and the hidden sources that support them [10]. Another limitation of ChatGPT’s medical ability lies within OpenAI’s usage policy that states to not provide tailored medical/health advice without review by a qualified medical professional [11]. Thus, medical information regarding one’s ED must be cautiously taken to avoid serious complications.

Lastly, the authors should be commended for their great efforts – albeit at its infancy stages—in evaluating the role of AI in ED. The integration of AI such as ChatGPT and BARD in healthcare delivery is still a controversial topic and indeed warrants further studies. However, to date, 1100 citations are referencing “ChatGPT” on PubMed showcasing the irreversible path to change [5]. Nonetheless, these AI models demonstrated excellent readability and accuracy when addressing the epidemiology and hazards of ED, they had difficulty in explaining the available alternatives for therapy and prevention, which may have limited their usefulness in managing ED in real-world settings. Moreover, concerns about lack of medical training and human-like delivery provide a risk of over-reliance and mistakes in medical queries, making physician participation in creation and review necessary to guarantee readability and correctness [10]. Furthermore, it would be useful to compare the levels of proficiency measured by each study in a standardized way to truly assess the accuracy of AI languages for ED issues. Finally, there is no doubt that the utilization of AI-based language machines may in part solve the advancement of urological care and the management of ED symptoms. However, the need for physician oversight and further studies assessing the efficacy of management of ED symptoms in clinical practice is necessary for further endorsement of AI models such as ChatGPT 3.5, ChatGPT 4, and BARD.
